# A Functional and Structural Investigation of the Human Fronto-Basal Volitional Saccade Network

**DOI:** 10.1371/journal.pone.0029517

**Published:** 2012-01-03

**Authors:** Sebastiaan F. W. Neggers, Rosanne M. van Diepen, Bram B. Zandbelt, Matthijs Vink, René C. W. Mandl, Tjerk P. Gutteling

**Affiliations:** Department of Psychiatry, Rudolf Magnus Institute of Neuroscience, UMC Utrecht, the Netherlands; Tokyo Medical and Dental University, Japan

## Abstract

Almost all cortical areas are connected to the subcortical basal ganglia (BG) through parallel recurrent inhibitory and excitatory loops, exerting volitional control over automatic behavior. As this model is largely based on non-human primate research, we used high resolution functional MRI and diffusion tensor imaging (DTI) to investigate the functional and structural organization of the human (pre)frontal cortico-basal network controlling eye movements. Participants performed saccades in darkness, pro- and antisaccades and observed stimuli during fixation. We observed several bilateral functional subdivisions along the precentral sulcus around the human frontal eye fields (FEF): a medial and lateral zone activating for saccades in darkness, a more fronto-medial zone preferentially active for ipsilateral antisaccades, and a large anterior strip along the precentral sulcus activating for visual stimulus presentation during fixation. The supplementary eye fields (SEF) were identified along the medial wall containing all aforementioned functions. In the striatum, the BG area receiving almost all cortical input, all saccade related activation was observed in the putamen, previously considered a skeletomotor striatal subdivision. Activation elicited by the cue instructing pro or antisaccade trials was clearest in the medial FEF and right putamen. DTI fiber tracking revealed that the subdivisions of the human FEF complex are mainly connected to the putamen, in agreement with the fMRI findings. The present findings demonstrate that the human FEF has functional subdivisions somewhat comparable to non-human primates. However, the connections to and activation in the human striatum preferentially involve the putamen, not the caudate nucleus as is reported for monkeys. This could imply that fronto-striatal projections for the oculomotor system are fundamentally different between humans and monkeys. Alternatively, there could be a bias in published reports of monkey studies favoring the caudate nucleus over the putamen in the search for oculomotor functions.

## Introduction

In primates, parallel excitatory and inhibitory recurrent loops from cortical motor areas through the basal ganglia (BG) are assumed to modulate cortical activity and preset the motor system for either volitional or automatic behavior [1]. This control system is often investigated using the antisaccade task [2], requiring simultaneous suppression of an automatic eye movement and execution of a volitional eye movement.

Most knowledge regarding oculomotor control arises from single cell recordings in non-human primates. The cortical frontal and supplementary eye fields (FEF, SEF) contain visual and saccade related neurons [3]. FEF saccade neurons activate for saccades directed contralaterally [4]. The FEF sends eye movement signals directly to the ipsilateral superior colliculus (SC) in the midbrain [5]. The SC provides access to the brainstem oculomotor nuclei driving the extraocular eye muscles [6]. The FEF and SC also contain so called ‘preparatory set’ neurons signaling task instruction even before a saccade target appears [7]. Besides projecting to the SC directly, the FEF is also connected to the SC through the striatum in the basal ganglia in several ways [8]. These pathways can inhibit or excite the SC, thus resolving conflicting situations. Oculomotor activation is usually observed in the monkey striatal subdivision caudate nucleus, where lateralization with respect to saccade direction is less clear [1]. Within the striatum the caudate nucleus is therefore referred to as ‘oculomotor striatum’ [1], as opposed to the putamen being the ‘skeletomotor striatum’ [9].

The above animal model is often used to explain and diagnose behavioral symptoms for brain pathologies affecting the basal ganglia. Although recent evidence from functional imaging points to the existence of similar networks in humans, a direct comparison with animal models is difficult due to poor spatial resolution and lack of structural connectivity data in human studies. In many conventional human neuroimaging studies the FEF is observed somewhere along a large strip coinciding along the precentral sulcus and premotor cortex [10], [11], [12], [13], [14], [15], [16]. Its precise location, however, varies substantially over studies due to different behavioral paradigms, acquisition techniques and analysis (mainly normalization approaches to a common stereotactic atlas space differ, if attempted at all). For example, using visually guided saccade tasks and fMRI, activation was observed in the superior section of precentral sulcus [11] Furthermore, the location of this visually guided saccade activation was found to co-vary consistently with the junction of the precentral sulcus and the superior frontal sulcus [12], [16]. However, during saccades executed in darkness using PET, activation was found along a clearly more lateral and inferior section of the precentral sulcus/premotor cortex [17]. There are numerous other studies defining the FEF in different ways. For example, using self-paced visually guided saccades and delayed pro and anti saccades, two foci of activation were observed along the superior precentral sulcus and more laterally in the premotor cortex/inferior precentral sulcus [18], [19]. In a study investigating memory guided saccades [13] activation was also observed in two similar foci, contralateralized with respect to saccade direction, as in monkeys. The same study observed largely overlapping contralateralized topographic maps for both memorized items without any saccades in the same two areas along the precentral sulcus. One focus was located at the junction of the precentral and superior frontal sulci as known to activate for visually guided saccades [12], and one more lateral along the inferior precentral sulcus. Interestingly, larger ‘preparatory set’ related activation following anti as compared to pro saccade task cues, but preceding the saccades themselves, has been observed in the superior section of the human precentral sulcus [20], [21] The reverse pattern as was observed for the monkey FEF [7].

Furthermore, using standard imaging resolution and visually guided saccades, antisaccades or saccades in darkness several human studies report oculomotor activation mainly in the putamen [22], [23], [17], [24], [25] that together with the caudate nucleus forms the striatum in the basal ganglia. However, oculomotor functions are generally reported for neurons in the caudate nucleus for monkeys. This discrepancy with the macaque animal model where the caudate nucleus is considered the oculomotor striatum warrants further investigation of the human striatum with different tasks on the same set of subjects. Furthermore, in contrast to monkey studies, ‘preparatory set’ signals have never been observed for the human striatum, but only for the human FEF [20], [21]. The human caudate nucleus, as in monkeys, seems sensitive to saccade control signals [26], but this seems related to a change in rather than an absolute level of ‘preparatory set’. As the FEF is supposedly connected to the striatum, it is worthwhile to search for preparatory set signals in the human striatum as well. Finally, how the striatum and supposed oculomotor subdivisions of the precentral sulcus are structurally interconnected is largely unknown so far. The fact that a number of human fMRI studies observed clear activation for saccade paradigms in the putamen casts fist doubt on the assumption that the human fronto-basal circuitery underlying eye movements is comparable to the oculomotor network as reported for monkeys.

Summarized, there is a clear need to measure precentral and striatal oculomotor activation maps with a set of behavioral tasks specifically aiming at a oculomotor and visual functions in the same individual subjects. Based on the discrepancies between the reported locations of the human FEF in studies using different behavioral paradigms we hypothesize that functional subdivisions of oculomotor function exist along the precentral sulcus. Also, the visual activation along the anterior precentral sulcus evoked by saccade stimuli alone, as is reported for monkeys [3], is not clear for humans. Furthermore, the pattern of structural connectivity between the frontal oculomotor structures and the striatum needs to be investigated to confirm the picture arising from the activation maps in these areas. To carefully map the fronto-basal network supporting human oculomotor function we investigated the FEF, SEF, striatum and midbrain in detail using 3T fMRI at a high resolution. Oculomotor functions were probed by saccades in darkness, visual sensory function by observing stimuli while maintaining fixation and volitional functions by comparing anti with prosaccades. Using DTI we investigated how the FEF and SEF functional subdivisions are connected to the striatum.

## Materials and Methods

### Participants

Thirteen healthy volunteers aged 20–35 participated in this study (7 male, 6 female). Written informed consent was obtained, and the procedures were approved by the Medical Ethical Committee of the University Medical Center Utrecht. Participants had no history of psychiatric or neurological disorders, and had normal or corrected to normal vision.

### Behavioral paradigms

**Figure 1:**
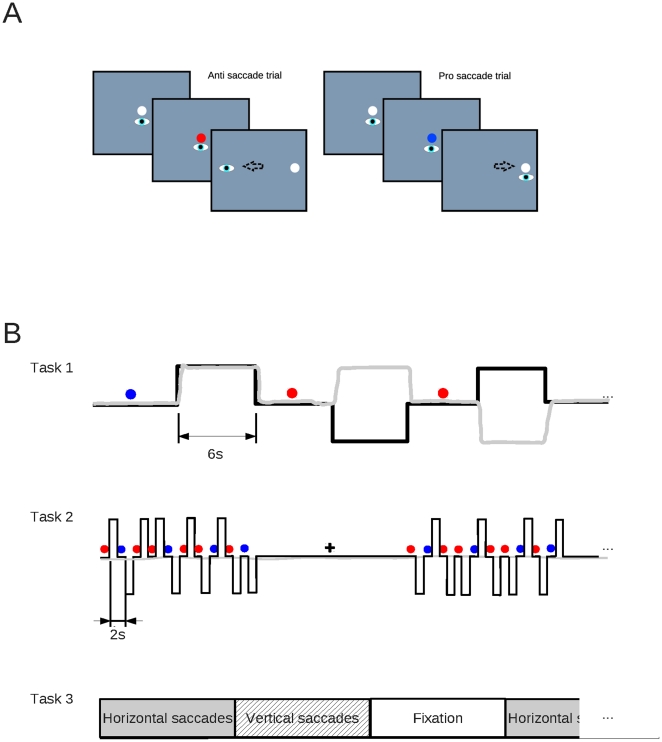
Outline of behavioral paradigms. A). The stimulus panels used in pro- and antisaccade trials. Panels drawn on the background in this figure are presented first. Instructed eye position as a reaction to each stimulus is indicated in the panels. In reality the targets were presented on a black background. B). Timelines with important events for all 3 tasks. For task 1 and 2, the solid black lines indicate the location of the eye movement target/fixation dot, and the gray solid lines indicate eye position. Colored circles indicate pro and antisaccade cues, and the black cross in the scheme for task 2 indicates a fixation epoch. For further details see the methods section.

Participants performed three tasks during fMRI scanning. The stimulus displays for the first two tasks were always pro- and antisaccade stimuli with two possible amplitudes (large and small), where the color of the central cue indicated whether to make a pro- or antisaccade. The rate of trial presentation and the instruction for the participants differed between task 1 and 2. For the stimulus displays used see figure 1. Stimuli were presented on a black background. Stimuli were projected on a 1×1 m^2^ screen hanging down from the ceiling, at about the level of the participant's feet. Subjects viewed the stimuli through a mirror mounted on the MR head coil. The effective viewing distance was 2 m.

Task 1 is designed as an event-related task and designed to detect activation induced when inhibiting an automatic eye movement and performing an instructed eye movement instead. Participants performed pro and antisaccades at a slow pace. In event related fMRI designs, one has to allow several seconds between events of interest for which one wants to separate and contrast activation [27]. This is due to the slow nature of the hemodynamic response that takes more than 10 seconds to return to baseline. In this study allowing sufficient time between events is important because we want to compare anti with prosaccade activation, antisaccade cues with prosaccade cues as well as account for variance in the signal induced by saccades back to the center. The task (trial 1) started at the beginning of the first fMRI volume by using a TTL trigger from the scanner. A trial started with a white fixation dot in the center (circle with 1 degree of visual angle). After three seconds this dot changed its color from white to red or blue, indicating whether a pro- or antisaccade had to be made as soon as a peripheral target appears. 3 seconds after the colored cue, the peripheral target (white circle with 1 degree of visual angle) appeared at 3.8 or 14.8 degrees of visual angle to the left or right, and the fixation dot disappeared. The two different amplitudes were chosen to prevent high predictability of saccade metrics and thus overlearning of saccade responses. In prosaccade trials, participants had to perform a saccade towards this target as soon as possible, and maintain fixation at this target for as long as it is visible. In antisaccade trials participants had to make a saccade to the empty location on the screen opposite to the location the peripheral target appeared. Whether the red/blue cues indicated a pro- or antisaccade was counterbalanced over participants. After 6 seconds the peripheral target disappeared and the central white fixation dot reappeared, indicating a saccade back to the center had to be made. After this a new trial starts. No rest period was included (the task ran non stop during each run). The time between subsequent saccades was on average about 6 seconds (see figure 1). The aforementioned presentation times of the fixation target, task cue and peripheral target are averages as a random time shift between +/−500 ms was added (‘jittering’) to each event to prevent predictability. This task was performed in three runs of 8 minutes each. Between runs the scanning and stimuli were halted and a brief pause was interleaved to allow the participants to briefly relax. Eye movements were not monitored as we know from previous studies using this or very similar tasks [10], [28] that hardly any erroneous prosaccades are made on the antisaccade or fixation trials (mainly due to the long time in between events). Subjects could briefly practice the task before scanning started, which is known to reduce the number of erroneous prosaccades to a small percentage, even in more difficult anti saccade paradigms [29].

Task 2 is designed to detect activation due to visual target presentation, but not saccade execution. The stimulus panels were identical to task 1, but participants were instructed to keep fixating the center of the screen throughout the run, no saccades were allowed. Furthermore, presentation rate was much faster: the fixation target and task cue were each visible for 500 ms, the peripheral target for 1 second. Also, stimulus presentation lasted 20 seconds after which participants had to fixate a white cross at the center of the screen (size 1×1 degree of visual angle and line thickness 0.1 degree of visual angle) for 20 seconds, constituting a rest epoch. This cycle was repeated 12 times, resulting in a scanning run of 8 minutes. Note that due to this difference in presentation rate the BOLD activation magnitudes will differ between tasks. Therefore, activation magnitude was never contrasted directly between tasks, but rather the differences in the location of task related activations are compared qualitatively (see data analysis). Different presentation rates were chosen as the main aim of this study is the qualitative comparison of functional zones within the FEF and other oculomotor areas in the human brain. Completely event related designs for all 3 tasks would have prolonged scanning time to over two hours, leading to serious fatigue for participants and hence unreliable performance.

Task 3 was designed to detect activation related to the execution of saccades. Participants were blindfolded such that the blindfold did not touch the eyelids. Subjects had to keep their eyes open as during normal viewing. During the first 20 seconds participants were asked to make self paced horizontal saccades from left to right at a rate of about one saccade/s. After that participants were asked to make vertical saccades at the same rate, from top to bottom. For the following 20 seconds participants were asked to keep fixating straight ahead with their eyes open, which served as the ‘rest’ period that activation during saccade blocks will be contrasted with (see data analysis). This cycle was repeated for 15 minutes. The task was indicated by an assistant touching the participants left foot, right foot or both, indicating horizontal saccade blocks, vertical saccade blocks or fixation.

### MR acquisition

**Figure 2:**
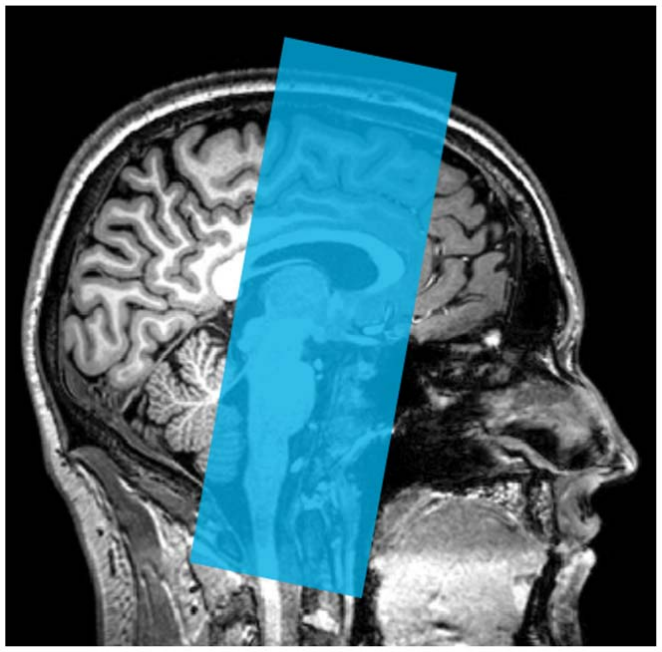
Field of view (FOV). The field of view used for fMRI acquisition during task performance, indicated as a blue translucent square overlaid on a sagittal slice through the anatomical scan of one of the participants. Care was taken that the striatum, midbrain, FEF and SEF were in the field of view. In between functional runs, the angulation was checked regularly and adjusted if required.

All volunteers were scanned in a Philips Achieva 3T scanner equipped with a 8 channel head coil allowing parallel imaging, while performing the three tasks described above. During task performance a high-resolution coronal gradient echo 2D-EPI fMRI sequence was used with a field of view covering the FEF, SEF, basal ganglia and midbrain (see figure 2). Acquisition parameters: GRE EPI, TR = 2100 ms, TE = 30 ms, flip angle = 78°, 128×1 28 matrix, 2×2 mm^2^ in-plane resolution, 30 coronal slices, phase encoding direction left-right, slice thickness 2 mm, FOV = 256*256*60 mm, SENSE factor R = 2. After each fMRI session a ‘whole brain EPI scan’ was recorded with the same parameters and in the exact same angulation as the 2D-EPI used during the tasks, but with 90 slices and hence a TR of 5461 ms such that it covered the entire brain. This scan was used for registration and normalization purposes of the fMRI data (see data analysis) for which the scan used during task performance was lacking coverage. Two DTI scans covering the entire brain were subsequently acquired: single shot EPI-DTI scan consisting of 30 diffusion-weighted scans (b = 1000 s/mm^2^) with non-colinear gradient directions and one average of five diffusion unweighted scans (b = 0 s/mm^2^), TR = 7035, TE = 68 ms, matrix 128×128, 1.875×1.875 mm^2^ in-plane resolution, 75 axial slices, phase encoding direction PA (AP 2^nd^ scan), slice thickness 2 mm, no slice gap, FOV 240×240×150 mm^3^, SENSE factor 3, EPI factor 35, no cardiac gating. DTI was measured twice with phase encoding direction reversed the second time in order to correct for susceptibility induced spatial distortions known to occur in such images (Andersson et al., 2003). Finally, a high resolution T1 weighted structural scan covering the whole brain was acquired : TR = 9.87 ms, TE = 4.6 ms, flip angle 8°, FOV 224×224×160 mm^3^, matrix 256×256, in-plane resolution 0.875×0.875 mm^2^, 200 axial slices, slice thickness 1 mm, no slice gap. This scan was used to register with the DTI data and normalize it to MNI space (see data analysis).

Cardiac signals were measured at 500 Hz with ECG electrodes and respiration with a belt wrapped around the waste. The electrocardiogram (ECG) and respiratory belt equipment built into the Philips Achieva scanner was used. This was done in order to remove of cardiac and respiratory pulsatility contaminating BOLD fMRI time series (see data analysis for details).

### Data analysis

#### fMRI

fMRI data analysis was done using SPM5 (http://www.fil.ion.ucl.ac.uk/spm/software/spm5) and matlab scripts developed in-house. Spatial preprocessing of the data and subsequent statistical modeling of fMRI time series analysis is described in detail below.

First, the raw fMRI data was spatially preprocessed. Images were realigned to correct for head motion in the scanner, using rigid body transformations, and a mean image was created. Next, the data were temporally interpolated per slice to correct for the individual timing differences in slice acquisition, such that the signal of each slice was interpolated to the time of acquisition of the middle slice. The ‘whole brain EPI scan’ was coregistered to the mean fMRI image created during realignment, and subsequently segmented using ‘unified segmentation’ [30]. Using the normalization parameters resulting from ‘unified segmentation’, the fMRI images were normalized to MNI template space. The latter procedure was chosen because rigid body coregistration of the fMRI time series images to the ‘whole brain EPI scan’ is close to perfect, due to the fact that the whole brain EPI scan has the exact same spatial distortions as the fMRI images acquired during task performance (angulation was kept identical, see ‘MR acquisition’).

Such distortions are known to occur in EPI imaging, especially for large acquisition matrices (ie, high resolution) as used here. Normalization includes non-linear local transformations and is thus well able to correct for these spatial distortions when transforming the fMRI images to MNI space. Coregistration of the fMRI images to a T1 weighted anatomical scan and then normalization of the T1 scan and fMRI images to MNI space, as is the common procedure, is difficult here due to the spatial deformations in the fMRI images. Finally the fMRI images were spatially smoothed with a gaussian kernel with a FWHM of 4 mm.

The preprocessed time series data for all tasks was subjected to a General Linear Model (GLM) analysis per participant (1^st^ level analysis).

#### Task 1

Data from task 1 was statistically modeled in a rapid event-related manner [27]. Two different GLM models were constructed. In the first model, 12 event types were defined and included to construct event related regressors; 8 events for the peripheral target presentation for leftward and rightward, small and large pro and antisaccades, and 4 event types representing target steps back to the center for leftward and rightward large and small target amplitudes (no difference was made between pro- and antisaccade trials for saccades back to the center). The events for target steps back to the center were only included to serve as ‘nuisance regressors’ that can regress out the hemodynamic responses (HRs) caused by saccades back to the center. Twelve functions of time (event-functions) representing each of these events were constructed using Dirac delta functions. These functions were convolved with the canonical HR function (HRF) as implemented in SPM5 (two gamma functions) and used as regressors in the GLM analysis. Three contrasts between conditions were computed for this analysis: between anti and prosaccades, and between leftward and rightward pro and antisaccades separately.

To test for task cue activation (the red or blue circle indicating that anti or prosaccades will have to be made), a 2^nd^ GLM model was constructed. In this model, 14 event types were defined and included to construct event related regressors; 2 events for the central cue indicating anti and prosaccades, and the same 12 events for the first GLM model described above. Otherwise the model was constructed similarly as the first GLM model. The contrast comparing anti with pro cues is computed.

#### Task 2

The preprocessed data from task 2 (visual stimulation during fixation) was modeled using a GLM with two task regressors. For the first regressor, a box-car function (with value 1 for 20 s during the saccade task and 0 during 20 s fixation epoch) was convolved with the default canonical hemodynamic response function from SPM5. The second regressor is a constant modeling baseline. A contrast testing the regression coefficient of the task regressor against zero was calculated (i.e. testing for higher activation during the stimulation blocks as compared to rest).

#### Task 3

The data from task 3 (saccades in darkness) was modeled with 2 HRF convolved box-car task regressors, for vertical and horizontal saccade blocks separately, and a constant regressor. The contrast testing the sum of vertical and horizontal saccade block regression coefficients against zero was computed, effectively testing which voxels exhibit significant activation during both saccade blocks as compared to the rest period (active fixation).

Finally, to all four 1^st^ level GLM models described above 20 ‘nuisance regressors’ were added to model cardiac and respiratory pulsatility, according to the RETROICOR method used with 5^th^ order fourier expansions [31]. The algorithm from Glover et al (2000) [31] was implemented in matlab functions developed in-house. The ECG and respiratory belt signals described in the previous section and acquired for all functional MRI runs were used for these purposes. Physiological non-neuronal rhythms are known to be massively present in BOLD data, especially in midbrain and basal ganglia areas due to, among others, the arterial circle of Willis vasculature. Modeling such rhythms as covariates using RETROICOR will increase sensitivity to neuronal activation of interest. Temporal autocorrelation in the fMRI data was modeled using autoregressive (AR) modeling of the first order by pre-whitening the GLM equation. Data was also high-pass filtered during pre-whitening with a cutoff cycle length of 128 s. Whitening hyperparameters were estimated per voxel during a first-pass GLM estimation. The latter is a common procedure in SPM5 and well described in part 3 of Statistical Parametrical Mapping [32].

All contrast images were subjected to a 2^nd^ level random effects one sampled t-test, testing for each contrast which voxels were significant on the group level. Connected clusters were considered to be significantly different from 0 when significant at p<0.05 on a cluster level, whole brain corrected. As a cluster defining threshold p<0.001 uncorrected was used, and a minimal extent threshold was determined with this cluster defining threshold using a Monte-Carlo simulation. As this extent threshold depends on residual variance, it differs slightly between tasks and is mentioned in table 1. Interesting activations at trend level (below this extent threshold, but with p>0.1 corrected) were sometimes included as well but clearly described as such.

**Table 1:** Details on the observed clusters of activation for contrasts from all three tasks are presented.

Area label	MNI coordinates (x,y,z)	T_max_	#voxels
Task 1: event related anti vs prosaccades (Ct = 14)			
Left ‘antisaccade FEF’	−26 −4 54	6.163	102
Right ‘antisaccade FEF’	30 −6 66	7.59	156
SEF left	−8 10 50	5.84	6
SEF right	4 6 58	4.26	2
PUT left anterior	−18 14 4	4.775	3
PUT right anterior	22 16 0	5.41	3
PUT right	22 −2 6	5.41	6
CN left	−16 −8 18	4.83	6
CN right	10 −2 14	4.34	2
Task 1: event related cue anti vs cue pro (Ct = 13)			
Left FEF	−32 −6 52	12.73	16
Right FEF	34 0 50	5.23	7
PUT right	22 16 2	5.67	10
PUT left	−28 −2 0	5.03	2
Task 2 (visual stimulation vs rest) (Ct = 14)			
SEF left	−8 6 52	8.15	111
SEF right	8 12 40	6.84	102
FEF left1	−38 −6 52	5.72	30
FEF left2	−52 0 38	6.42	54
FEF right	44 −2 52	8.81	375
Task 3 (saccades in darkness vs. fixation) (Ct = 17):			
Left ‘lateral FEF’	−54 0 40	5.80	26
Right ‘lateral FEF’	54 −2 36	6.29	37
Left ‘medial FEF’	−38 −14 44	4.68	6
Right ‘medial FEF’	44 −4 52	6.77	55
SEF left	−4 −4 64	10.12	50
SEF right	4 −4 58	5.48	19
PUT left	−26 −2 0	7.18	104
PUT right	24 4 −2	5.62	31
SC/SNpr/RN	4 −28 −10	6.60	23
Thalamus left	−8 −22 −2	5.44	2
Thalamus right	10 −18 4	5.49	14

From left to right, columns represent the label for each area as used throughout the paper, the MNI coordinates of the voxel in each cluster with maximal T-value, the maximum T-value and the number of voxels in each cluster. The contrast for which the clusters are listed are named on top of each list of activations. At the right side of each contrast name the minimum cluster size is given for a cluster to be considered significant at the group level. That is, clusters with a lower cluster size are statistical trends. Trends are presented for the sake of completeness, and only discussed further when confirmed by significant findings from PSTH analysis (see figure 5). The quoted regions ‘lateral and medial FEF’ and ‘antisaccade FEF’ are also referred to as such in the text and further explored in the PSTHs, and where labeled such for sake of simplicity. Technically it is of course debatable whether all regions could be considered part of a single human FEF region. In general, when a label contains ‘FEF’ it is meant that it was located somewhere along the precentral sulcus or premotor cortex. Abbreviations: FEF: frontal eye fields, SEF: supplementary eye fields, PUT: putamen, CN: caudate nucleus, SC: superior colliculus, SNpr: substantia nigra pars reticulata, RN: red nucleus.

#### PSTH timecourses

For a selection of activation clusters (regions of interest or ROIs) at the group level observed for the tasks described above, the Peri-Stimulus Time Histogram (PSTH) will be calculated as the activation time course per condition, for the saccade types from the event related task (task 1). This can be thought of as averaged event-related responses corrected for overlap of subsequent overlapping events, which is sometimes referred to as ‘selective averaging’. In order to determine a PSTH, a Finite Impulse Response (FIR) model was applied to the onset of each target stimulus and was fitted to the time series data of each voxel, and as such one can compute BOLD timeseries without assuming any predetermined shape of the hemodynamic response [33]. The regression coefficients of a FIR model constitute the PSTH and reflect the averaged BOLD signal for a series of time points, here aligned at target stimulus onset. As saccade latencies are typically less than 300 ms and typical BOLD responses last about 10 seconds, PSTH alignment would have been largely identical when modeled at saccade onset. PSTHs for left- or rightward pro or anti saccades were computed separately (FIR window length 12 s and bin size 0.500). Data for large and small saccades is pooled. To remove low frequency signal drifts, high pass filtering was performed alongside with the FIR modeling using a 128 s cut off frequency. The PSTHs were created with in-house matlab scripts, making use of matlab functions available in the SPM5. Note that antisaccades to the left were defined as a saccadic eye movement towards the left, and hence a target located at the right side of the screen. The above procedure was repeated for every participant, and PSTHs were averaged over participants and all voxels in an activation cluster of interest.

### DTI

#### DTI image preprocessing

DTI image preprocessing was performed with software developed in-house [34]. All subsequent registration steps of fMRI activation images and fiber coordinates as well as fiber selection with ROIs were done in matlab scripts developed in-house using SPM5 Matlab functions, among others.

The DTI data set was simultaneously realigned to correct for head motion and corrected for possible EPI distortions. The DTI data set was corrected for such spatial distortions caused by susceptibility artifacts by exploiting the fact that DTI was scanned twice and with reversed phase encoding direction [35]. A robust estimation of the diffusion tensors was obtained using M-estimators [36] to limit the influence of possible outliers. From the diffusion tensors an FA image was calculated [37]. The T1-weighted structural scan was coregistered (using a rigid body transformation and the normalized mutual information implementation of SPM5) to the diffusion-unweighted image (B0), and therewith to the images containing the estimated diffusion tensors.

The T1-weighted image was segmented into white matter, gray matter and cerebral spinal fluid using unified segmentation [29] in SPM5. The normalization parameters (flow fields) resulting from this algorithm were used to normalize the DTI fiber tracts to MNI atlas space on a later stage of processing (see below). Furthermore, the normalized T1 weighted structural scans were averaged over all participants to create an image to overlay fMRI activations on for display purposes, as it represents the true anatomical spatial precision at the group level. Therefore, no clear sulci can be seen in this average anatomical image, representing the somewhat limited precision of the inter-subject anatomical match accomplished by most normalization procedures.

#### Fiber tracking

Fiber tracking was performed using an implementation of the FACT [38] using in-house developed software [34]. All possible fibers from every 2×2×2 mm^3^ voxel to each other voxel were tracked, resulting in a large series of polygons (x,y,z coordinates) representing fiber tracts. The following parameters were used: 8 seed points per voxel, minimum FA = 0.2, maximum angle = 53 degrees and maximum average angle with neighboring voxels = 90 degrees. Then all reconstructed tracts (i.e. the sets of x,y,z coordinates) were normalized to MNI space. As described before the required normalization parameters were derived from segmenting of the T1 weighted structural scan, that was coregistered with the tensor images and hence the fiber tracts.

The complete set of DTI fibers per participant was subjected to further processing steps as described below and depicted in the results section on DTI. We first set out to isolate the fibers connecting with the human caudate nucleus and putamen. To that order, we manually segmented the left and right caudate nucleus and putamen for each participant individually based on their normalized T1 weighted scans using the ROI tools in Mricron (http://www.cabiatl.com/mricro/mricron). We also dilated these ROIs with 2 mm. This prevented that fiber bundles diverging in the vicinity of a target region, potentially leading to lower FA values, result in a failure to detect fibers due to the minimum FA criterium (0.2) mentioned above. Using these four masks, from the complete set of fibers resulting from brute force tracking we selected those fibers running through these masks and have a fractional anisotropy (FA) lower than 0.3 at the intersecting location. The latter FA criterion, on top of the brute force fibertracking FA criterion mentioned above, implicates the fiber terminates (or starts) at the putamen or caudate, as the diffusion at that location is close to a minimum. Without the latter FA criterion tracts running from the cortex to areas more inferior than the striatum, but closely skimming the striatal surface, would be counted as connected to the striatum, contaminating our selected bundles. Each fiber in the four sets is then prolonged 3 times at both ends by 2 mm per step, by taking the average direction of the last 4 vectors of the fiber per step, resulting in an effective smooth prolongation of 6 mm. This was done to extend fibers running to the gray matter of the cortex, as DTI fiber tracking tends to stop closely before reaching gray matter due to decreasing FA. Finally, fibers coming from a mask and running across the midsagittal plane are cut at the midsagittal plane. The four resulting sets of fibers for each mask (left and right caudate nucleus and putamen) are saved to disk per participant.

With these sets of fibers we created a 3D group probability map indicating the likelihood of a fiber from the left and right caudate nucleus and putamen running through a certain location, to determine whether a global pattern of connectivity exists with certain parts of the cortex. That is, individual MNI space binary maps (i.e. maps with 1 s and zeros) were first created for each of the 4 bundles, by tagging every voxel in a 2×2×2 grid over the entire brain with 1 when a fiber from the MNI space bundle was running through it. This created 4 binary maps per participant, one for each ROI (left and right caudate nucleus and putamen). These binary maps were subsequently dilated with 3 voxels (ie 6 mm) and smoothed with 4 mm FWHM, and then averaged over participants to create a rough estimate of the probability of a bundle running through a certain location.

Finally, with the four sets of fiber bundles originating in the left and right caudate nucleus and putamen we determined the likelihood at which clusters of fMRI activations observed in the FEF and SEF are connected to the caudate nucleus or putamen (see results for which clusters). These fMRI activation clusters were determined individually per participant and task and saved to disk. The individual clusters of activated voxels were dilated by 4 mm to reflect some of the fiber tracking inaccuracies, and labeled according to their vicinity to the group level peak activations as presented in table 1. By doing so we could reliably isolate and label individual locations of activation, that were still close enough to the group results in the present paper. These individual areas of activation were then used to track DTI fibers to. Finally, we constructed ‘connectograms’ reflecting the probability of a connection between each cortical fMRI cluster and the striatal ROIs by simply counting how many participants (out of all) showed any connection at all.

## Results

### Included participants

We could analyze all data for 12 out of the total of 13 participants. For one participant, the quality of the DTI data and the fMRI data during task 2 and 3 was too low, mainly due to excessive head movement. For this participant, we only used the data from task 1 (event related pro and antisaccade task). Therefore, in the results below the number of participants was 12 except for the results from task 1, where data from all 13 participants could be used.

### fMRI

**Figure 3:**
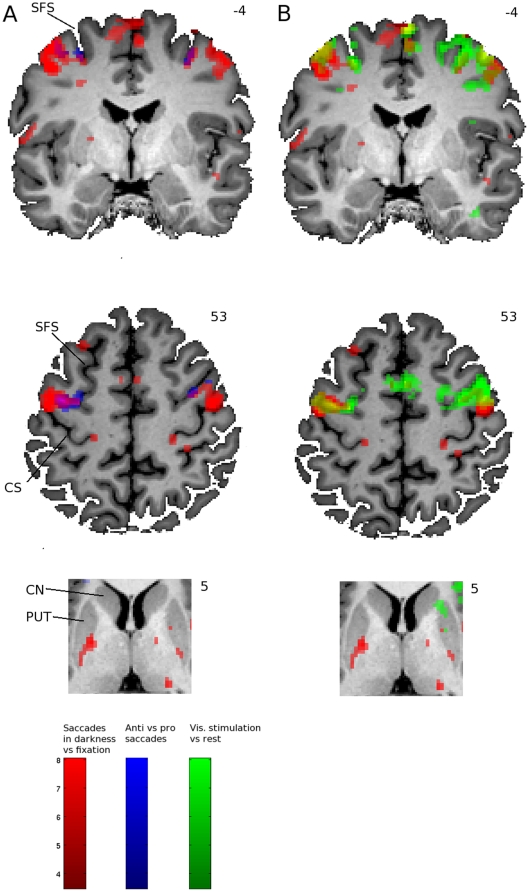
Single participant activation maps. The activation patterns (T-maps) are presented for a representative participant. In the left column (A), a coronal and 2 axial slices are shown with activation during saccades in darkness vs rest in red (task 3) and antisaccades as compared to prosaccades in blue (task 1). In the right column (B) the same slices are shown again with activation for saccades in darkness vs rest in red (task 3) and visual activation during fixation vs rest in green (task 2). Activation maps are overlaid on slices from the normalized anatomical scan of the same subject. In the upper 2 slices activation in the FEF and SEF can be seen, and in the lower slice a zoomed in section of the striatum where saccade activation is observed in the putamen. MNI coordinates of the slices is given in upper right hand side of each slice (y-coordinate for coronal slice, z-coordinate for axial slices). Activation is thresholded at T = 3.5. Slices are displayed in neurological convention (left = left). Labels: SFS: superior frontal sulcus; CS: central sulcus; PUT: Putamen; CN: Caudate Nucleus.

In figure 3 the activation maps for all three tasks are presented for a representative participant, overlaid on slices from the normalized anatomical scan of that participant. In the anterior medial precentral sulcus a patch of activation (rendered in blue) exhibits larger activation for anti as compared to prosaccades during the event related task (task 1), extending deep into the posterior portion of the superior frontal gyrus. Along the anterior precentral sulcus a large patch of visual activation during fixation (task 2) was observed, as well as in a separate cluster in the medial wall of the cortex. It seemed to overlap with the smaller region preferentially activating for antisaccades in task 1. Furthermore, slightly posterior from the visual and anti saccade activation, a clear patch of lateral and medial activation for saccades made in darkness vs rest (task 3) can be observed bilaterally (rendered in red) along the precentral sulcus and premotor cortex, and in a separate region along the medial wall of the cortex (probably the human SEF) slightly posterior from the visual activation from task 2.

Interestingly, the posterior putamen was clearly and bilaterally activated for saccades in darkness. No such activation was observed in the caudate nucleus.

**Figure 4:**
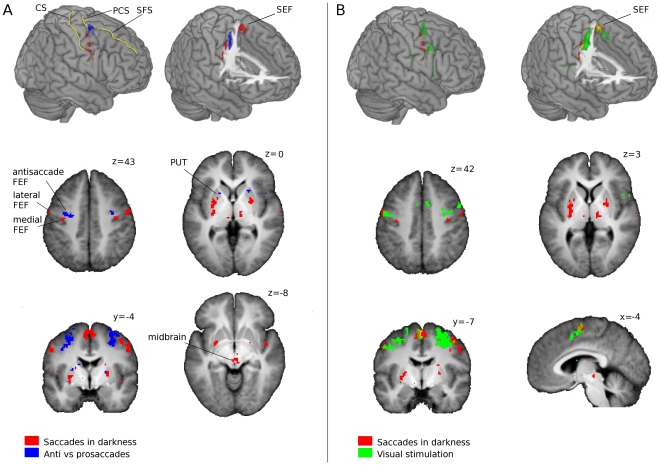
Group activation maps. Overview of group activation patterns (T-maps) for three tasks. The figure was split into panels A and B as combining activation overlays obscured too much detail due to overlap. Panel A at the left shows event related anti vs prosaccade activation in blue and saccades in darkness activation in red. Panel B at the right shows activation for visual stimulation vs rest in green and again for saccades in darkness vs rest in red. Overlap is rendered in yellow. In the upper row of panel A and B group activation is rendered in 3D on top of a high-quality individual (MNI normalized) skull stripped brain, only to indicate the location of activation with respect to the main sulci and gyri. In the right 3D rendering in panels A and B the anterior upper right part of the brain is cut out to show activation in deeper sulci and along the medial wall of the cortex. In the lower rows activation is overlaid on selected 2D slices through the T1 weighted normalized anatomical scan averaged over all participants, providing a more realistic impression of anatomical precision after normalization. Slice MNI coordinates are given for each slice at the upper right hand side (z-coordinate for axial slices, y for coronal and x for sagittal slices). Activation for all renderings is thresholded at T = 3.5, implying that also some stronger trends are displayed for the sake of completeness. See table 1 for an overview of statistics. Slices are displayed in neurological convention (left = left). Labels are indicating regions of interest or sulci. Lateral and medial FEF (frontal eye fields): two foci activated during saccades in darkness vs rest; antisaccade FEF: preferentially activated for antisaccades vs prosaccades (note that FEF label is only used for sake of simplicity; lateral activations are probably not part of the human FEF homologue). SEF: supplementary eye fields; PUT: Putamen; CS: central sulcus; SFS: superior frontal sulcus; CS: central sulcus.

At the group level similar observations were made (see figure 4), implying that the individual activations depicted in figure 3 are indeed representative for all scanned participants and the population from which they were drawn. The labeled foci and their MNI coordinates are presented in table 1. Along the precentral sulcus and premotor cortex, the region where most studies define the human FEF, lateral and medial bilateral clusters are consistently observed for saccades in darkness. Again, visual activation during fixation (task 2) was observed in a larger strip slightly anterior from the activation for saccades in darkness (task 3). Slightly anterio-medial from the medial cluster activated for saccades in darkness, a specific bilateral cluster that is more active for anti as compared to prosaccades was observed, extending deep into the junction of the precentral and superior frontal sulci. This activation was largely overlapping with activation occurring for visual stimulation during fixation (task 2), as can be seen in figure 4. From all voxels along the precentral sulcus and medial wall (a manually created mask was used encompassing these areas) exhibiting activation that was larger for anti as compared to prosaccades, 49% overlapped with voxels exhibiting activation for visual stimulation during fixation (task 2). It should be noted that the location of anti-saccade related activation is inferred from sulci of an individual brain normalized to stereotactic MNI space (top rows in figure 4). In an average of MNI normalized anatomical T1 weighted scans from the 13 current participants (as used in panels in the lower rows of figure 4) the common precentral and superior frontal sulci are hardly visible due to the current impossibility to exactly match individual sulci during normalization. Still, the sulci of the individual participants are close to the individual sulci depicted in figure 4, and labeling of observed clusters of activation should be reliable.

In the remainder of the results section and figures, the precentral regions activated for saccades in darkness will be referred to as ‘lateral and medial FEF’ and the cluster more active for anti as compared to prosaccades as ‘antisaccade FEF’. Note that this labeling is only introduced for sake of simplicity and readability, and not as a definitive functional subdivision. Technically it is of course debatable whether all regions observed could be considered part of a single human FEF region, especially whether the ‘lateral FEF’ can be considered part of the human FEF (see discussion).

Along the medial wall of the cortex an area was bilaterally activated for saccades in darkness, which could well be the human homologue of the SEF region in non-human primates. In the following we will refer to this area as the SEF.

Subcortically, in the putamen we observed a bilateral cluster of activation for saccades in darkness. In the caudate nucleus, often referred to as the ‘oculomotor striatum’ in non-human primates, we found no significant activation for saccades in darkness. Interestingly, in the anterior putamen we observed a bilateral cluster (at the trend level) that is activated more for anti as compared to prosaccades. Furthermore, a region in the midbrain extending from the deep layers of the SC to the red nucleus, periaquaductal gray and substantia nigra was activated during saccades in darkness. Finally, bilateral activation for saccades in darkness was observed at the trend level in the inferior bilateral thalamus.

Note that at the group level the activation clusters are more focal than at the individual level. This is most likely due to the fact that we scanned at a resolution that is relatively high for fMRI at 3T (2×2×2 mm^3^, where 4×4×4 mm^3^ is still common). As the anatomical overlap between the participant's brains is not perfect due to limitations inherent to normalization procedures and due to the fact that a perfect structure-function relationship does not exist in a comparable fashion over participants, we might mainly have observed the significant peak of functional representations that overlapped for most participants.

The following activation clusters of connected voxels for the group level contrasts described above were extracted as regions of interest (ROIs; images with 1 s for activated voxels and 0 s otherwise): left and right SEF, ‘lateral FEF’, ‘medial FEF’ and putamen for saccades in darkness vs rest (task 3) and ‘antisaccade FEF’ for anti vs prosaccades (task 1).

**Figure 5:**
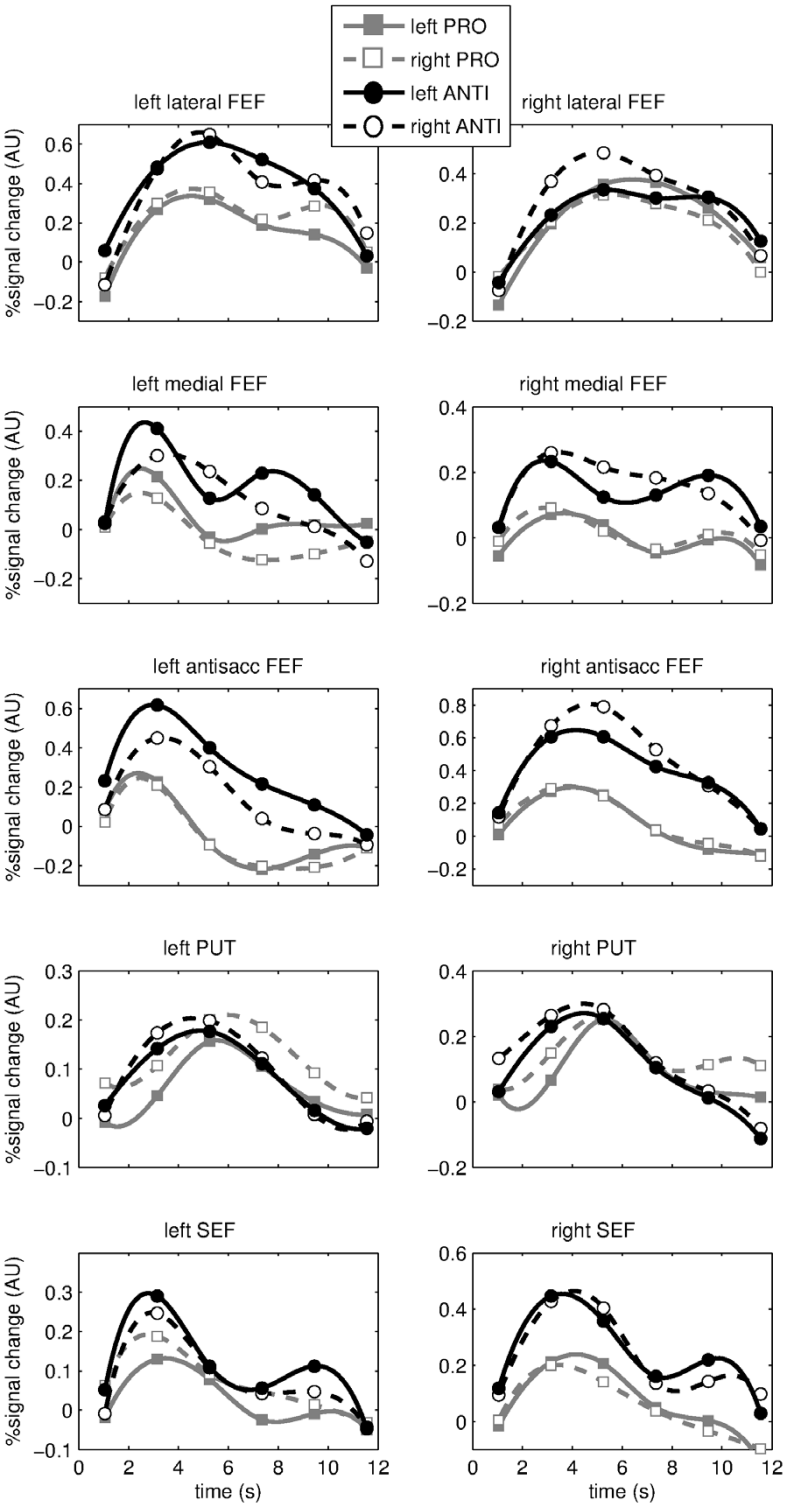
ROI activation timecourse. The average peri-stimulus-time-histograms (PSTHs; BOLD time courses) are shown for several ROIs: the clusters activated for saccades in darkness vs rest in left and right precentral sulcus/premotor cortex (‘lateral and medial FEF’) and the left and right putamen and SEF, and the left and right clusters in the FEF more active for anti as compared to prosaccades (‘antisaccade FEF’). The aforementioned group activation patterns from which the ROIs were taken are depicted in figure 4. PSTHs were averaged over voxels in the ROIs and then participants, and aligned at target stimulus onset. Saccade onset typically follows within 300 ms, therefore PSTHs aligned at saccade onset would have been largely identical. Data for the left hemisphere are given in the left panels and data for the right hemisphere in the right panels. In each panel, average PSTHs are presented for pro and antisaccades (gray squares and black circles) to the left and right (solid and dashed lines with open and solid symbols). The unit on the ordinate is global % signal change. The absolute magnitude of this unit is not directly meaningful and should not be compared over regions, as averaging took place over different numbers of voxel per region and within different brain areas. Absolute BOLD measures are known to vary considerably over regions. Differences between conditions within a region can be compared. Except the bilateral putamen, the right ‘lateral FEF’ and left SEF, peak activation in all ROIs was significantly larger for antisaccades as compared to prosaccades (p<0.05). The left SEF exhibited larger responses for antisaccades at trend level, T(12) = 1.59;p = 0.06. Further tests on single timepoints of interest are presented in the text of the results section.

To further investigate the timecourse of BOLD activation following target presentation in the event related task (task 1), we computed peri-stimulus-time-histograms (PSTHs) for voxels in the ROIs described before. PSTHs indicate the average BOLD timecourse aligned at target stimulus onset and were computed separately for leftward and rightward pro and antisaccades to investigate lateralization with respect to saccade direction and antisaccade preference. PSTHs were averaged over voxels within each cluster of interest and over participants. Average PSTHs are displayed in figure 5.

Clearly, PSTH amplitudes are larger for anti as compared to prosaccades in most of the areas of interest, except the bilateral putamen, the right ‘lateral FEF’ and left SEF. This was confirmed with post-hoc t-tests on the PSTH values at the sample time point around the peak of the PSTHs, averaged over all voxels in an ROI and leftward and rightward saccades (all ROIs from figure 5 except the aforementioned tested significant at p<0.05). Note that the left SEF exhibited larger responses for antisaccades at trend level, T(12) = 1.59;p = 0.06. The difference between anti- and prosaccades was largest in the ‘antisaccade FEF’, which is not surprising as this region is selected from a statistical contrast comparing anti with prosaccades. Interestingly, also the left lateral FEF zone and right SEF exhibited a preference for antisaccades in their PSTHs, which probably did not survive more stringent statistical testing at the voxel level as was done in figure 4. Furthermore, one can clearly see that the ‘antisaccade FEF’ shows larger activation for antisaccades directed to the ipsilateral side, that is, the PSTH amplitude is larger for leftward antisaccades in the left ‘antisaccade FEF’ and larger for rightward saccades in the right ‘antisaccade FEF’ ROI. This lateralization effect was tested statistically for PSTH amplitudes at the 2^nd^ and 3^rd^ timepoint (3.15 s and 5.25 s marker symbols in figure 5) as follows: the difference between PSTH amplitudes for leftward and rightward antisaccades in the left ‘antisaccade FEF’ was tested against the difference between PSTH amplitudes for leftward and rightward antisaccades in the right ‘antisaccade FEF’, using a 2-sampled t-test. When this value is maximal, lateralization with respect to antisaccade direction is maximal. This measure was found to be significantly different for both timepoints (T(12) = 3.78;p<0.005 at 3.15 s and T(12) = 3.48;p<0.005 at 5.25 s) in the left ‘antisaccade FEF’ and right ‘antisaccade FEF’ ROI pair. Such an effect was not observed for any other ROI pair.

**Figure 6:**
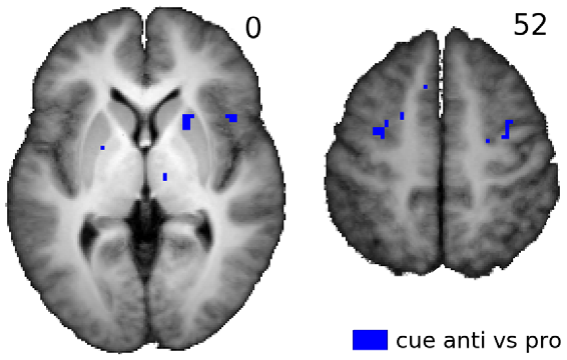
Cue evoked activation maps. Two axial slices with group activation patterns (T-maps) for the second GLM analysis are shown comparing antisaccade cue induced vs prosaccade cue induced activation (from task 1) in blue. Data is overlaid on the T1 weighted anatomical scan averaged over all participants. The z-coordinates (MNI space) is given for each slice at the upper right hand side. Activation is thresholded at T = 3.5. Slices are displayed in neurological convention (left = left).

The putamen and right lateral FEF region activated for saccades in darkness are the only areas where antisaccades did not evoke larger responses than prosaccades, not even at the trend level. However, in the putamen antisaccades appear to evoke BOLD responses somewhat earlier than prosaccades, as can be seen in figure 5. This was further investigated statistically. First, indicative post-hoc t-tests comparing the PSTH level at the 2^nd^ and 3^rd^ sample point (at the 3.15 s and 5.25 s markers in figure 5) were performed. At the 3^rd^ sample point the PSTH peaks and its rising flank occurs around the 2^nd^ sample point. PSTH amplitude at the 2^nd^ sample point was significantly higher for antisaccades as compared to prosaccades (T(12) = 2.03;p<0.05; left and right PUT were pooled), but at the 3^rd^ sample around the peak this was not the case (T(12) = 0.15). The latter is a first indication that the rising flank of the PSTH occurs earlier for antisaccades. This finding of earlier antisaccade activation in the putamen might very well be caused by the task cue presented on average 3 s before the onset of the peripheral target and saccade towards it. Namely, the cue could give rise to increased activity for upcoming antisaccades, thus implementing ‘preparatory set’. To further investigate where cue-related effects are located, the activation induced by the cue was also modeled and tested statistically at the voxel level. To this end, a new GLM analysis was run as described in the methods section. This analysis models cue related activation for anti and prosaccade cues separately. The contrast between anti and prosaccade cue induced activation was computed. Voxels testing positive for this contrast indicate areas where the cue evoked larger activation for anti as compared to prosaccades. As the onset of peripheral targets themselves is also modeled, and hence signal variations due to targets/saccades are explained, the voxels testing positive for the latter contrast cannot be activated by temporally overlapping saccade related responses rather than cue related responses. The areas where cue evoked activation was larger for antisaccades as compared to prosaccades are shown in figure 6, peak activations and MNI coordinates given in table 1. Interestingly, the left ‘antisaccade FEF’ activated significantly for anti as compared to prosaccade cues, the right ‘antisaccade FEF’ and parts of the left and right putamen at trend level. This trend was clearest in the right putamen.

### DTI fibertracts connecting the fronto-striatal oculomotor network

**Figure 7:**
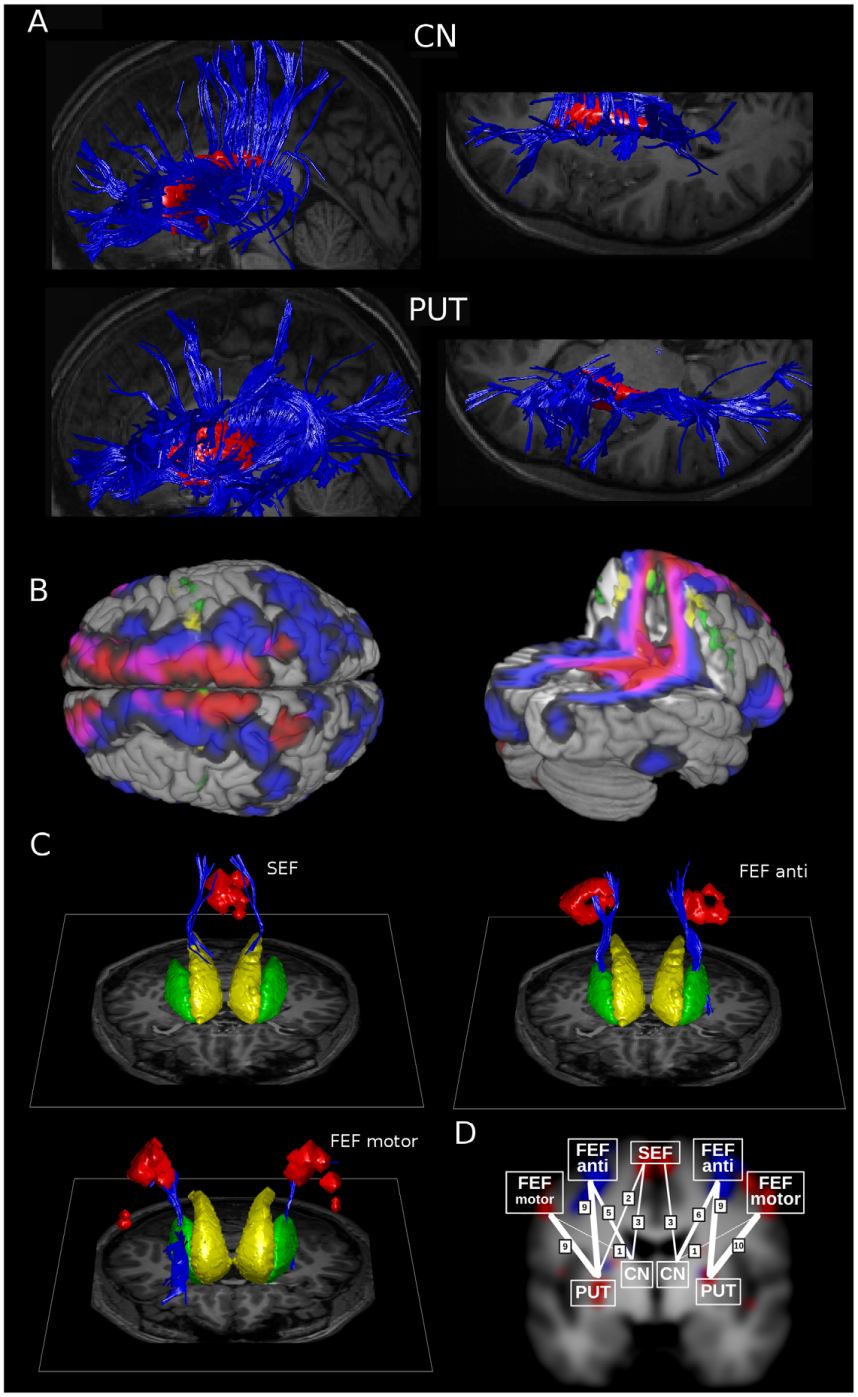
Step-wise results of DTI analysis. The subsequent steps in DTI fiber analysis are outlined in 4 panels. In panel A, the normalized fiber tracts originating from the left and right putamen and caudate nucleus are rendered in blue for one participant together with orthogonal slices from the normalized T1 weighted scan. The manually segmented ROIs for the caudate nucleus and putamen are also rendered in red. Panel B shows a 3D representation of the average probability (over participants) for a voxel to be connected to the caudate nucleus (in red) or putamen (in blue) or both (purple). In yellow the zones activated for antisaccades and in green the zone activated for saccades in darkness are rendered. For each participant, it was then investigated which fibers from the 4 fiber bundles originating in the left and right putamen and caudate nucleus were connected to the most important cortical fMRI activation clusters observed in 4. See panel C for example results from individual participants: fibers connecting the caudate nucleus and SEF are shown in the left rendering, fibers connecting the putamen and antisaccade zone in the FEF in the right rendering, and the lower left rendering shows fibers connecting the areas activated for saccades in darkness (‘lateral and medial FEF’) with the putamen. The manually segmented caudate nucleus and putamen are also shown in yellow and green, respectively. Panels C is presented in order to illustrate the fiber processing steps, and are not necessarily representative. Panel D shows for how many participants (out of 12) regions were connected at all. ‘FEF motor’ refers to zones along the precentral sulcus activated for saccades in darkness (that is, ‘lateral FEF’ and ‘medial FEF’ taken together), ‘FEF anti’ refers to zones activated for antisaccades vs prosaccades. Line thickness also indicates the number of subjects with connections. The diagram is overlayed onto a blurred slice from figure 4, only to roughly indicate the location.

DTI data was processed in several steps: putamen and caudate nucleus masks were manually segmented in all participants, fibers were tracked from these masks, and finally connections to FEF and SEF subdivisions as determined with fMRI were counted (for details see methods). Figure 7 shows representative results from these steps. In panel A, the normalized fiber tracts originating from the left and right putamen and caudate nucleus masks are rendered for one participant together with orthogonal slices from the normalized T1 weighted scan of the same participant. The caudate nucleus and putamen masks are also shown. For this participant it can be clearly seen that the caudate nucleus tends to be connected to the medial-most parts of the entire anterior cortex, from the central sulcus to the frontal cortex. The putamen is connected to more lateral regions along the entire cortical sheet.

Panel B shows a 3D representation of the average probability (over participants) for a voxel to be connected to the caudate nucleus (in red) and putamen (in blue) or both (purple). It can be seen that the observations for the single participant in panel A are representative for the group of participants. That is, the caudate nucleus is mainly connected to the medial part of the anterior half of the cortex, and the putamen to a more lateral band spanning the entire cortex.

For each participant we investigated which fibers from the four bundles originating in the left and right putamen and caudate nucleus were connected to the most important individual cortical fMRI activation clusters : the precentral zones observed for saccades in darkness (that is the ‘lateral FEF’ and ‘medial FEF’ taken together, dubbed ‘motor FEF’ in figure 7), the ‘antisaccade FEF’ activating preferentially for antisaccades, and the SEF zone activated for saccades in darkness. When there was at least one fiber running between a striatal ROI (left and right caudate nucleus and putamen) and a cortical activation cluster, it was counted as connected for that participant. Note that the fMRI activation clusters used as DTI seed were determined individually for this analysis. See panel C for results for 1 participant: fibers connecting the caudate nucleus and SEF are shown in the left rendering and fibers connecting the putamen and antisaccade zone in the FEF in the right rendering of panel C. The manually segmented caudate nucleus and putamen are also shown.

Panel D shows for how many participants (out of 12) regions were ‘connected’ at all. From panel D it can be seen that the ‘motor FEF’ zones activated for saccades in darkness were most clearly connected to the putamen, and hardly to the caudate. The ‘antisaccade FEF’ was also preferably connected to the putamen, but also exhibited connections to the caudate nucleus for a considerable number of participants. The SEF was most clearly connected to the caudate nucleus, but the number of participants for which a connection with either the caudate nucleus or putamen was observed at all was rather low. This is probably due to the smaller size of the SEF activation as compared to the FEF, and the particular location of the SEF near the medial wall. We will therefore not quantitatively compare the connectivity of the SEF with the striatum to connectivity of the FEF, as it would be biased towards the FEF. Comparing connectivity of parts of the FEF with the striatum is fair as their relative size is comparable, as are its relative distances to both the caudate nucleus and putamen.

## Discussion

In the present study, we used high resolution 3T fMRI and DTI to investigate function and structure of the human fronto-basal saccade network. First the human oculomotor areas around the precentral sulcus (FEF), along the medial wall (SEF) and striatum were investigated in detail by presenting visual stimuli during fixation, self paced saccades in darkness and during a pro and antisaccade task. Second, we investigated how the network was interconnected using DTI fiber tracking. Finally, we determined how this network reacts to a cue indicating the upcoming task, thus implementing preparatory set.

In the cortex the oculomotor area along the precentral sulcus (FEF) exhibited clear bilateral functional subdivisions for saccades in darkness, anti saccades and visual stimulation. For saccades in darkness, two small bilateral areas were observed along the lateral premotor cortex and medial precentral sulcus. These activations correspond well to the location of the FEF as observed for saccades in darkness using PET [17]. This saccade related activation could be related to the saccade neurons that can actually evoke saccades, as reported for monkeys in the posterior FEF [3]. As can be seen in figure 5, visually guided pro and antisaccades also evoked activation in the medial and lateral clusters activated for saccades in darkness, probably due to the motor component needed to produce pro and antisaccades. It is important to note that activation for saccades in darkness is located along the lateral precentral sulcus and premotor cortex, and not the lateral postcentral gyrus. Namely, it was recently observed that proprioception signals from extraocular muscles is represented along the lateral part of the human central sulcus [39], but clearly more posterior than the activation for saccades in darkness reported here.

Similar activation for saccades in darkness was also observed in the medial wall of the cortex (human homologue of SEF). This activation overlaps with an earlier study mapping SEF activation in 6 individuals using saccades in darkness [40], observing the SEF along the upper part of the paracentral sulcus. Our average SEF coordinates are slightly (less than 1 cm) more anterior and superior, and we consistently observed SEF bilaterally rather than largely unilaterally as in Grosbras et al. (1999). Our SEF coordinates also match well to the coordinates reported in a study with 10 subjects using visually guided saccades [13]. The aforementioned study used a design with multiple saccade directions in rapid succession and compared activation to a baseline rest epoch; study design and saccade frequency were similar to our saccades in darkness task.

Slightly anterio-medial from the medial area along the precentral sulcus activating for saccades in darkness, a bilateral area preferentially activated for antisaccades was observed, extending deep into the precentral sulcus and most posterior portion of the superior frontal sulcus. This was the only region showing lateralization with respect to saccade direction: activation was larger for antisaccades directed ipsilaterally. No such lateralization was observed for prosaccades. Slightly anterior of the SEF area activated for saccades in darkness, a cluster activating preferentially for antisaccades was observed as well (trend). This spatial separation of antisaccade preferring areas from areas activated for saccades in darkness near the precentral sulcus (FEF) and the medial wall (SEF) has, to our knowledge, not been reported before. When pooled over all voxels in the medial FEF zone activated for saccades in darkness, there was a slightly larger response for antisaccades as well (see figure 5), probably because this zone is in close vicinity to and somewhat overlapping with the region preferentially activated for antisaccades. This difference between anti and prosaccades was not significant on the voxel level within the region activated for saccades in darkness.

An extensive strip activating for visual stimulus presentation during fixation was observed slightly anterior from the medial and lateral precentral areas activated for saccades in darkness. This visual activation pattern was largely overlapping with the more focal area preferentially activated for antisaccades. A similar visual activation cluster was observed in the anterior SEF. Again, this spatial separation of visually evoked activation from the activation for saccades in darkness along a anterior-to-posterior axis near the precentral sulcus and SEF has, to our knowledge, not been reported before. The extensive overlap between the visual activation for task 2 and the smaller area preferentially activated for antisaccades in task 1 could implicate an overlap in function. Namely, during active fixation saccades to the salient onset of a target need to be suppressed, similar to when producing antisaccades. Alternatively, the FEF is involved in controlling shifts of visual attention towards peripheral stimuli [14], which probably occurs during the fixation task as well as during the antisaccade task. It is virtually impossible to prevent such shifts in covert attention when peripheral stimuli appear during visual fixation. At the very least, the FEF seem to contain posterior areas activated for saccades in darkness, and anterior areas activated during the presentation of visual stimuli, the inhibition of saccades and/or visual attention shifts. Also note that is not possible here to directly contrast visual activation from task 2 with event-related anti or prosaccade activation from task 1 due to the different presentation rates used in both taks (see methods section). However, the location of activation in both tasks and the fact that they overlap is meaningful with respect to underlying function.

The area preferring antisaccades at the junction of the precentral sulcus and superior frontal sulcus corresponds well with other studies that defined the FEF as a region activated during visually guided pro or antisaccades [10], [11], [12], [14], [19], [16] as can be concluded from comparing the MNI space coordinates reported in table 1 of the present study with the MNI space coordinates in the aforementioned studies. It can be debated whether the most lateral area activated for saccades in darkness is still to be labeled FEF, as it is quite far from the junction of the precentral and superior frontal sulci typically associated with human FEF using visually guided saccades. However, it does activate for saccades in darkness and slightly anterior of this lateral saccade region visual activation was observed, similar to more medial activation foci.

The different types of activation observed here along the precentral sulcus and premotor cortex agree well with the visual, movement and anticipatory neurons found in the monkey FEF [41], [3]. Monkey visual FEF neurons also seem located slightly anterior to saccade neurons in the anterior bank of the arcuate sulcus [3], [42]. However, for monkeys this division is not clear cut nor replicated by others, and based on only a few monkeys.

Larger activation for antisaccades in the entire FEF were also reported in other human imaging studies [23], [20], [43], [11], [19], [27], whereas we observed it in a smaller subdivision of the FEF. This is probably due to the fact that we used a relatively high acquisition resolution and improved spatial normalization techniques providing a better match between the brain shapes of individual participants and hence less blurring in activation maps at the group level [29], [44].

Contrary to the reports on human FEF activation as measured with fMRI, FEF neurons in monkeys are actually less active during antisaccades than during prosaccades [7]. A recent study showed that monkeys scanned with fMRI exhibit increased FEF activation for antisaccades as well [45], implying that the discrepancy with human FEF activation reports appeared to be related to methodological differences between fMRI and extracellular single cell recording rather than differences between species. The latter report suggests that this can be explained by the fact that in the FEF, fMRI BOLD reflects mostly activation due to the metabolism of neuronal input to FEF neurons and FEF interneurons, whereas single cell recordings are biased to the larger pyramidal cells. Also note that others did not find overall different activation in FEF neurons for pro- and antisaccades towards singleton stimuli in a search array [46]. Congruent with the above, one should always exert caution comparing magnitudes and even the sign of neuronal activation as measured with fMRI and single cell recording; BOLD fMRI and single neuron recording are different but complementary measurements of brain activation. BOLD is in general more sensitive to metabolic processes occurring around synaptic transmission of neuronal signals [47], [48], and single cell recording measures the action potentials (‘spikes’) along the axon or soma.

Furthermore, we observed a preference for ipsiversive antisaccades (i.e. larger activation for leftward/rightward antisaccades in the left/right FEF, see PSTHs in figure 5) in the antisaccade zone along the medial precentral sulcus, whereas in monkeys FEF saccade neurons prefer contraversive antisaccades [7]. This could be explained by the aforementioned difference between fMRI and electrophysiology: when antisaccades evoke larger responses in BOLD fMRI, but the opposite in single cell recording, the lateralization with respect to antisaccade direction is probably also reversed. Therefore the lateralization observed here could imply that the antisaccade zone suppresses the automatic prosaccade, enabling a correct antisaccade. Alternatively, the presentation of the target stimulus could explain why antisaccades evoke larger BOLD responses and the preference for ipsiversive saccades. For antisaccades the stimulus is in the opposite hemifield as the direction of the saccade, and therefore ipsiversive antisaccade preference could as well be contralateral visual preference, known to exist in non-human primate visual FEF neurons [3]. Furthermore, the target stimulates the retina twice: before and after a saccade. Therefore, the response to the presaccadic contralateral stimulus in visuomotor FEF neurons with an eccentric (i.e. non-foveal) receptive field superimposed with activation caused by the same but even more eccentric post-saccadic contralateral target stimulus would yield larger responses for antisaccades than prosaccades. Namely, for prosaccades presaccadic activation is eccentric and post-saccadic activation foveal. The observed lateralization might therefore have nothing to do with suppressing prosaccades. The latter account for our results would also explain why another report where the peripheral target was extinguished did observe a small preference for contralateral antisaccades [19]. However, when the stimuli in antisaccade trials resulted in ipsilateralized FEF activation with respect to saccade direction in the present study, one would then have expected FEF activation contralateralized with respect to prosaccade direction, which we did not observe. However, this could be a statistical power issue as prosaccade activation in the FEF is generally smaller than antisaccade activation. The current results therefore cannot clearly distinguish between both explanations.

Finally, one would have expected contralateralized activation in the FEF for prosaccades, as is known to exist in the monkey FEF [4] and human FEF as revealed by electroencephalography [49]. Only some fMRI studies revealed a rather small preference for contralateral (pro) saccade direction in the FEF [19], [15], [50], whereas by far most fMRI studies investigating saccades do not report the expected lateralization. Possibly, this difference is not clear enough to pickup easily with BOLD fMRI. The most notable exception is a recent study showing clear contralateralized maps for saccade direction in a medial and lateral area along the precentral sulcus [13], corresponding well to the two bilateral zones of the FEF we observed here. Alternatively, several TMS studies demonstrated that in controlling visual attention the human FEF exhibits considerable hemispheric specialization [51], [52], indicating that perhaps lateralization with respect to saccade direction is also less strict than in monkeys.

In the basal ganglia, the bilateral putamen clearly activated for saccades in darkness. At the trend level, the anterior putamen activated preferentially for antisaccades. Interestingly, for antisaccades, activation in the putamen zone activating for saccades in darkness seemed to lead prosaccade activation in time. A cluster in the left medial precentral sulcus, near the ‘medial FEF’ antisaccade area we observed, revealed larger activation following the pre-target cue signaling antisaccade instructions as compared to prosaccade instructions. Similar effects were observed in the right FEF and anterior putamen at the trend level (see figure 6). This ‘preparatory set’ activation probably prepares the oculomotor system to inhibit an automatic eye movement to the salient target, and instead execute a volitional saccade to a region in space dictated by the task. Two other studies using longer intervals between the cue and the saccade target observed similar cue induced effects in the FEF, where an antisaccade cue elicited enhanced activation that remained elevated until the saccade was made [20], [21]. In our study the cue-target interval was shorter (3 s on average), but as this interval was jittered it will explain variance in the BOLD signal independent of the overlapping target evoked activation. Similar antisaccade cue activation has been reported for the FEF in monkeys using single cell recordings [7]. Recently in the monkey caudate nucleus [53] antisaccade cue induced activation was observed, but to our knowledge the present study is the first demonstrating antisaccade cue related activation in the human striatum, indicating that the fronto-putamen pathway plays a role in presetting the oculomotor system to inhibit an automatic saccade and execute a volitional saccade instead. Recently, it appeared that the human putamen is also involved in presetting the skeletomotor system to inhibit a prepared finger movement, indicating this function of the putamen is not limited to the oculomotor system [54], [55], [56], [57]. Interestingly, in contrast to antisaccade cue related activation in the putamen, cue related activation in FEF seems to remain elevated (see figure 5) even after antisaccade execution. This might imply that the FEF stay in some kind of ‘anti saccade’ setting even after the anti saccade has been produced, whereas pathways through the putamen signal whether an antisaccade is allowed or not, and returns to baseline after the saccade is made. Further research is needed to shed light on the underlying reason for this discrepancy.

Other subcortical locations activating for saccades in darkness include a region in the midbrain encompassing the deep layers of the SC, the red nucleus, periaquaductal gray and substantia nigra. The SC and substantia nigra are well known to be involved in saccade generation in non-human primates [8], but activation unambiguously related to saccade execution (and not visual stimulation) is nevertheless reported in only very few human functional imaging studies [10]. This is probably due to their small size and the fact that cardiac and respiratory noise (which we removed, see methods) are dominant around the midbrain. It is hard to distinguish between the aforementioned closely spaced midbrain areas, as due to inter group averaging and smoothing fMRI activation patterns could have merged. Finally, bilateral activation for saccades in darkness was observed at the trend level in the inferior bilateral thalamus. The thalamus also is a node in the recurrent fronto-striatal circuits controlling eye movements, relaying processed signals back to the FEF [8].

DTI fiber tracking revealed that the caudate nucleus is preferably connected to the fronto-medial cortex, and the putamen to a more lateral band spanning the entire cortex, as has been observed before [58]. The zones along the precentral sulcus found to be activated for saccades in darkness were connected preferably to the putamen, and the antisaccade zones both to the putamen and caudate nucleus with a clear preference for the putamen. Note that we will not further compare SEF with FEF connectivity as this comparison might be overly biased by their size and location on the cortical sheet. In fact, we could not establish much connectivity between the SEF and any of the regions of interest in the striatum. The pattern of connectivity between the striatum and the FEF observe here is in agreement with the observed fMRI activation for saccades in darkness in the putamen but not in the caudate nucleus. Furthermore, the human premotor cortex, in close proximity to the FEF, is also primarily connected to the putamen [59]. This pattern is clearly in disagreement with single cell studies performed on macaque monkeys [8], considering the caudate nucleus the ‘oculomotor striatum’. The putamen is generally considered the ‘skeletomotor striatum’ [9], [60], regulating responses for bodily movements other than eye movements. However, besides the present study two other human imaging studies investigating saccades while obtaining PET [17] and 7T fMRI data [25] also observed activation mainly in the putamen but not caudate nucleus. Other studies using different eye movement paradigms observed, among other activated areas, clear fMRI activation in the putamen as well [22], [23], [24]. Interestingly, a study investiging 9 patients with bilateral lesions to the putamen observed an increase in saccade errors and accuracy for memory guided saccades and self paced saccade sequences (comparable to our task using self-paced saccades in darkness), but for visually guided pro or anti saccades no differences in latency or the amount of errors was observed in comparison to matched healthy control subjects [61].

The human caudate nucleus has been found to play a role in eye movement control [10], [62]. This doesn't seem to be related, however, to pure saccade execution or even preparatory set, but rather seems to signal a change in preparatory set over time [26]. At least, when investigating basic saccade execution and suppression as in the present study, the putamen is clearly more involved than the caudate nucleus, and hence the term ‘oculumotor striatum’ for the human caudate nucleus as well as ‘skeletomotor striatum’ for the putamen should be reconsidered. From the present study and other published reports it seems that both parts of the striatum, the caudate nucleus and the putamen, are not so much subdivided according to which effector is controlled (e.g. the eye or arm/hand), as was previously assumed. Rather, the amount of executive control one has over a planned movement seems to determine whether the caudate nucleus or putamen is involved, regardless of the effector that is used. That is, when one has to pre-plan, inhibit or initiate movements or movement sequences, the putamen is involved, and when online executive control or a change in stimulus-response setting is required, the caudate nucleus seems more involved. Some authors favored similar accounts of the function of the caudate nucleus and putamen based on their findings [23], [63]. In general, it is nowadays accepted that the structural and functional organization of the striatum follows a rostro-caudal and dorso-ventral gradient [64]. The rostro-ventral striatum is primarily connected to the medial frontal, orbitofrontal and lateral frontal cortex, subserving functions as reward anticipation, whereas caudo-dorsal striatum receives inputs mainly from the premotor, motor, and parietal cortex, suggesting an important role in motor control in general. We suggest a further functional subdivision within the caudo-dorsal striatum with respect to the level of executive control over movements that an actor has, as describe before. Note that it is very well possible that this distinction also translates to behavioral control of functions other than the motor system. For example, language and memory are also strongly associated with caudate function [65], [66].

The current findings and the latter inferred scheme for striatal functional subdivisions could imply that the human oculomotor control network is organized fundamentally different as in non-human primates regarding striatal control signals. Alternatively, the oculomotor functions of the monkey putamen might not have been thoroughly investigated using single cell recording, as planned electrode sites are often chosen based on previous reports that most likely indicated the caudate nucleus as an area of interest for oculomotor function. Actually, studies injecting anterograde tracers in monkey FEF neurons as identified by single cell stimulation evoking saccades, observed partial putamen labeling, besides clear labeling in the caudate nucleus [67]. Therefore, a bias in monkey oculomotor studies of the striatum seems a more likely explanation why our findings differ from what was reported for monkeys.

The present study is to our knowledge one of the first investigating visual, saccade and preparatory functions and the anatomical connectivity of the human oculomotor system at this level of spatial detail within a single study. The functionally different oculomotor and visual subdivisions along the precentral sulcus and premotor cortex could together be considered the human ‘FEF complex’. The distinction between functional subregions of the human FEF complex as observed here can serve to disambiguate discussions about the human FEF homologue in future studies. Most aspects of the human ‘FEF complex’ are resembling findings from non-human primates, whereas human cortico-striatal anatomical connectivity and striatal activation patterns exhibit important discrepancies with the prevailing animal model of cortico-basal oculomotor control networks. This could be due to differences in the organization of fronto-striatal networks between humans and monkeys, or to the fact that investigations in monkeys are biased to the caudate nucleus.

## References

[1] Watanabe M, Munoz DP (2010). Presetting basal ganglia for volitional actions.. J Neurosci.

[2] Hallett PE (1978). Primary and secondary saccades to goals defined by instructions.. Vision Res.

[3] Schall JD (1991). Neuronal activity related to visually guided saccades in the frontal eye fields of rhesus monkeys: comparison with supplementary eye fields.. J Neurophysiol.

[4] Bruce CJ, Goldberg ME, Bushnell MC, Stanton GB (1985). Primate frontal eye fields. II. Physiological and anatomical correlates of electrically evoked eye movements.. J Neurophysiol.

[5] Sommer MA, Wurtz RH (2000). Composition and topographic organization of signals sent from the frontal eye field to the superior colliculus.. J Neurophysiol.

[6] Sparks DL (2002). The brainstem control of saccadic eye movements.. Nat Rev Neurosci.

[7] Everling S, Munoz DP (2000). Neuronal correlates for preparatory set associated with pro-saccades and anti-saccades in the primate frontal eye field.. J Neurosci.

[8] Munoz DP, Everling S (2004). Look away: the anti-saccade task and the voluntary control of eye movement.. Nat Rev Neurosci.

[9] Alexander GE, Crutcher MD, DeLong MR (1990). Basal ganglia-thalamocortical circuits: parallel substrates for motor, oculomotor, “prefrontal” and “limbic” functions.. Prog Brain Res.

[10] Neggers SFW, Raemaekers MAH, Lampmann EEL, Postma A, Ramsey NF (2005). Cortical and subcortical contributions to saccade latency in the human brain.. Eur J Neurosci.

[11] Ford KA, Goltz HC, Brown MRG, Everling S (2005). Neural processes associated with antisaccade task performance investigated with event-related FMRI.. J Neurophysiol.

[12] Amiez C, Kostopoulos P, Champod A, Petrides M (2006). Local morphology predicts functional organization of the dorsal premotor region in the human brain.. J Neurosci.

[13] Kastner S, DeSimone K, Konen CS, Szczepanski SM, Weiner KS (2007). Topographic maps in human frontal cortex revealed in memory-guided saccade and spatial working-memory tasks.. J Neurophysiol.

[14] Neggers SFW, Huijbers W, Vrijlandt CM, Vlaskamp BNS, Schutter DJLG (2007). TMS pulses on the frontal eye fields break coupling between visuospatial attention and eye movements.. J Neurophysiol.

[15] Ikkai A, Curtis CE (2008). Cortical activity time locked to the shift and maintenance of spatial attention.. Cereb Cortex.

[16] Amiez C, Petrides M (2009). Anatomical organization of the eye fields in the human and non-human primate frontal cortex.. Prog Neurobiol.

[17] Dejardin S, Dubois S, Bodart JM, Schiltz C, Delinte A (1998). PET study of human voluntary saccadic eye movements in darkness: effect of task repetition on the activation pattern.. Eur J Neurosci.

[18] Darby DG, Nobre AC, Thangaraj V, Edelman R, Mesulam MM (1996). Cortical activation in the human brain during lateral saccades using EPISTAR functional magnetic resonance imaging.. Neuroimage.

[19] Curtis CE, Connolly JD (2008). Saccade preparation signals in the human frontal and parietal cortices.. J Neurophysiol.

[20] DeSouza JFX, Menon RS, Everling S (2003). Preparatory set associated with pro-saccades and anti-saccades in humans investigated with event-related FMRI.. J Neurophysiol.

[21] Brown MRG, Goltz HC, Vilis T, Ford KA, Everling S (2006). Inhibition and generation of saccades: rapid event-related fMRI of prosaccades, antisaccades, and nogo trials.. Neuroimage.

[22] Petit L, Orssaud C, Tzourio N, Salamon G, Mazoyer B (1993). PET study of voluntary saccadic eye movements in humans: basal ganglia-thalamocortical system and cingulate cortex involvement.. J Neurophysiol.

[23] O'Driscoll GA, Alpert NM, Matthysse SW, Levy DL, Rauch SL (1995). Functional neuroanatomy of antisaccade eye movements investigated with positron emission tomography.. Proc Natl Acad Sci USA.

[24] Simó LS, Krisky CM, Sweeney JA (2005). Functional neuroanatomy of anticipatory behavior: dissociation between sensory-driven and memory-driven systems.. Cereb Cortex.

[25] Krebs RM, Woldorff MG, Tempelmann C, Bodammer N, Noesselt T (2010). High-field FMRI reveals brain activation patterns underlying saccade execution in the human superior colliculus.. PLoS ONE.

[26] Cameron IGM, Coe BC, Watanabe M, Stroman PW, Munoz DP (2009). Role of the basal ganglia in switching a planned response.. Eur J Neurosci.

[27] Burock MA, Buckner RL, Woldorff MG, Rosen BR, Dale AM (1998). Randomized event-related experimental designs allow for extremely rapid presentation rates using functional MRI.. Neuroreport.

[28] De Weijer AD, Mandl RCW, Sommer IEC, Vink M, Kahn RS (2010). Human fronto-tectal and fronto-striatal-tectal pathways activate differently during anti-saccades.. Front Hum Neurosci.

[29] Hodgson TL, Golding C, Molyva D, Rosenthal CR, Kennard C (2004). Reflexive, symbolic, and affective contributions to eye movements during task switching: response selection.. J Cogn Neurosci.

[30] Ashburner J, Friston KJ (2005). Unified segmentation.. Neuroimage.

[31] Glover GH, Li TQ, Ress D (2000). Image-based method for retrospective correction of physiological motion effects in fMRI: RETROICOR.. Magn Reson Med.

[32] Friston K (2007). Statistical parametric mapping : the analysis of funtional brain images. 1e ed.

[33] Goutte C, Nielsen FA, Hansen LK (2000). Modeling the haemodynamic response in fMRI using smooth FIR filters.. IEEE Trans Med Imaging.

[34] Mandl RCW, Schnack HG, Luigjes J, van den Heuvel MP, Cahn W (2010). Tract-based analysis of magnetization transfer ratio and diffusion tensor imaging of the frontal and frontotemporal connections in schizophrenia.. Schizophr Bull.

[35] Andersson JLR, Skare S (2002). A model-based method for retrospective correction of geometric distortions in diffusion-weighted EPI.. Neuroimage.

[36] Chang L, Jones DK, Pierpaoli C (2005). RESTORE: robust estimation of tensors by outlier rejection.. Magn Reson Med.

[37] Basser PJ, Pierpaoli C (1996). Microstructural and physiological features of tissues elucidated by quantitative-diffusion-tensor MRI.. J Magn Reson B.

[38] Mori S, Crain BJ, Chacko VP, van Zijl PC (1999). Three-dimensional tracking of axonal projections in the brain by magnetic resonance imaging.. Ann Neurol.

[39] Balslev D, Albert NB, Miall C (2011). Eye muscle proprioception is represented bilaterally in the sensorimotor cortex.. Hum Brain Mapp.

[40] Grosbras MH, Lobel E, Van de Moortele PF, LeBihan D, Berthoz A (1999). An anatomical landmark for the supplementary eye fields in human revealed with functional magnetic resonance imaging.. Cereb Cortex.

[41] Bruce CJ, Goldberg ME (1985). Primate frontal eye fields. I. Single neurons discharging before saccades.. J Neurophysiol.

[42] Suzuki H, Azuma M (1983). Topographic studies on visual neurons in the dorsolateral prefrontal cortex of the monkey.. Exp Brain Res.

[43] Matsuda T, Matsuura M, Ohkubo T, Ohkubo H, Matsushima E (2004). Functional MRI mapping of brain activation during visually guided saccades and antisaccades: cortical and subcortical networks.. Psychiatry Res.

[44] Crinion J, Ashburner J, Leff A, Brett M, Price C (2007). Spatial normalization of lesioned brains: performance evaluation and impact on fMRI analyses.. Neuroimage.

[45] Ford KA, Gati JS, Menon RS, Everling S (2009). BOLD fMRI activation for anti-saccades in nonhuman primates.. Neuroimage.

[46] Sato TR, Schall JD (2003). Effects of stimulus-response compatibility on neural selection in frontal eye field.. Neuron.

[47] Hyder F, Patel AB, Gjedde A, Rothman DL, Behar KL (2006). Neuronal-glial glucose oxidation and glutamatergic-GABAergic function.. J Cereb Blood Flow Metab.

[48] Logothetis NK, Pauls J, Augath M, Trinath T, Oeltermann A (2001). Neurophysiological investigation of the basis of the fMRI signal.. Nature.

[49] Gutteling TP, van Ettinger-Veenstra HM, Kenemans JL, Neggers SFW (2010). Lateralized frontal eye field activity precedes occipital activity shortly before saccades: evidence for cortico-cortical feedback as a mechanism underlying covert attention shifts.. J Cogn Neurosci.

[50] Beurze SM, de Lange FP, Toni I, Medendorp WP (2009). Spatial and effector processing in the human parietofrontal network for reaches and saccades.. J Neurophysiol.

[51] Grosbras M, Paus T (2003). Transcranial magnetic stimulation of the human frontal eye field facilitates visual awareness.. Eur J Neurosci.

[52] Silvanto J, Lavie N, Walsh V (2006). Stimulation of the human frontal eye fields modulates sensitivity of extrastriate visual cortex.. J Neurophysiol.

[53] Ford KA, Everling S (2009). Neural activity in primate caudate nucleus associated with pro- and antisaccades.. J Neurophysiol.

[54] Vink M, Kahn RS, Raemaekers M, van den Heuvel M, Boersma M (2005). Function of striatum beyond inhibition and execution of motor responses.. Human brain mapping.

[55] Vink M, Ramsey NF, Raemaekers M, Kahn RS Striatal dysfunction in schizophrenia and unaffected relatives.. Biol Psychiatry.

[56] Zandbelt BB, Vink M (2010). On the role of the striatum in response inhibition.. PloS one.

[57] Zandbelt BB, van Buuren M, Kahn RS, Vink M (in press). Reduced Proactive Inhibition in Schizophrenia Is Related to Corticostriatal Dysfunction and Poor Working Memory.. Biol Psychiatry.

[58] Leh SE, Ptito A, Chakravarty MM, Strafella AP (2007). Fronto-striatal connections in the human brain: a probabilistic diffusion tractography study.. Neurosci Lett.

[59] Draganski B, Kherif F, Klöppel S, Cook PA, Alexander DC (2008). Evidence for segregated and integrative connectivity patterns in the human Basal Ganglia.. J Neurosci.

[60] DeLong MR, Alexander GE, Georgopoulos AP, Crutcher MD, Mitchell SJ (1984). Role of basal ganglia in limb movements.. Hum Neurobiol.

[61] Vermersch AI, Müri RM, Rivaud S, Vidailhet M, Gaymard B (1996). Saccade disturbances after bilateral lentiform nucleus lesions in humans.. J Neurol Neurosurg Psychiatr.

[62] Raemaekers M, Jansma JM, Cahn W, Van der Geest JN, van der Linden JA (2002). Neuronal substrate of the saccadic inhibition deficit in schizophrenia investigated with 3-dimensional event-related functional magnetic resonance imaging.. Arch Gen Psychiatry.

[63] Hikosaka O, Wurtz RH (1985). Modification of saccadic eye movements by GABA-related substances. II. Effects of muscimol in monkey substantia nigra pars reticulata.. J Neurophysiol.

[64] Haber SN, Knutson B (2010). The reward circuit: linking primate anatomy and human imaging.. Neuropsychopharmacology.

[65] Ben-Yakov A, Dudai Y (2011). Constructing realistic engrams: poststimulus activity of hippocampus and dorsal striatum predicts subsequent episodic memory.. J Neurosci.

[66] Friederici AD (2006). What's in control of language?. Nat Neurosci.

[67] Stanton GB, Goldberg ME, Bruce CJ (1988). Frontal eye field efferents in the macaque monkey: I. Subcortical pathways and topography of striatal and thalamic terminal fields.. J Comp Neurol.

